# Deep resequencing of the voltage-gated potassium channel subunit *KCNE3 *gene in chronic tinnitus

**DOI:** 10.1186/1744-9081-7-39

**Published:** 2011-09-07

**Authors:** Philipp G Sand, Berthold Langguth, Tobias Kleinjung

**Affiliations:** 1 Department of Psychiatry, University of Regensburg Universitätsstr. 84, 93042 Regensburg, Germany; 2 Department of Otorhinolaryngology, University of Regensburg Franz-Josef-Strauss-Allee 11, 93053 Regensburg, Germany; 3 Department of Otorhinolaryngology, University of Zurich, Frauenklinikstrasse 24, 8091 Zurich, Switzerland

## Abstract

Membrane-stabilizing drugs have long been used for the treatment of chronic tinnitus, suggesting an underlying disturbance of sensory excitability due to changes in ion conductance. The present study addresses the potassium channel subunit gene *KCNE3 *as a potential candidate for tinnitus susceptibility. 288 Caucasian outpatients with a diagnosis of chronic tinnitus were systematically screened for mutations in the *KCNE3 *open reading frame and in the adjacent region by direct sequencing. Allele frequencies were determined for 11 known variants of which two (F66F and R83H) were polymorphic but were not associated with the disorder. No novel variants were identified and only three carriers of R83H were noted. However, owing to a lack of power, our study can neither rule out effects of *KCNE3 *on the risk for developing chronic tinnitus, nor can it exclude a role in predicting the severity of tinnitus. More extensive investigations are invited, including tests for possible effects of variation in this ion channel protein on the response to treatment.

## Findings

Tinnitus is an unpleasant sensation often described as 'ringing in the ears' that may manifest in a variety of settings, e.g. after acoustic trauma, as a side effect of medication, or spontaeously [1]. Epidemiological studies suggest that close to 15% of the adult population may be affected to varying degrees [2]. In severe cases, patients develop a chronic course of illness marked by sleep disturbances, depressed mood, increased muscle tension and loss of attention, or other comorbidities [3]. While the underlying biological mechanisms are still incompletely understood, early research into the pharmacological treatment of tinnitus has emphasized a role for cellular ion regulation and transport [4]. Interest in the disruption of ion conductance in the inner ear has been renewed following recent discoveries in other heritable pathologies of auditory perception. Specifically, dysfunctional K^+^-extruding cells of the stria vascularis are known to interfere with potassium homeostasis and the endocochlear potential in Jervell and Lange-Nielsen Syndrome (JLNS) types 1 and 2 [5,6], in DNFA2 [7], and in EAST syndrome [8], among others. Variants in the genes encoding voltage-gated potassium channel proteins KCNQ1, KCNQ4, and KCNE1 have also been proposed as candidate risk modifiers in more common disorders of the auditory pathway, e.g. in Menière's disease [9], in noise-induced hearing loss [10,11], in age-related hearing loss [12], and in chronic tinnitus [13].

A further candidate gene, encoding the potassium channel ß subunit KCNE3/MIRP2, has been adressed in Menière's disease [9]. Like KCNE1, *KCNE3 *is expressed in the mammalian inner ear [14] and brain [15,16]. Both proteins interact to regulate trafficking, surface expression, and activation of another potassium channel, KCNH3, in the cortex and in other parts of the central nervous system [17]. So far, a limited number of investigations has addressed sequence variation in *KCNE3 *which maps to chromosome 11q13-14, a linkage hot-spot for autosomal recessive, non-syndromal hearing impairment [18]. In view of the frequent association of tinnitus and hearing impairment [19], and of its cooccurence with Menière's disease [20], we hypothesized that tinnitus may be part of the phenotypic spectrum that is caused by *KCNE3 *variants.

In 288 outpatients (202 men and 86 women, age 50.1 ± 12.6 yrs, mean ± SD) consulting for chronic tinnitus (minimum duration of 6 months), the diagnosis was confirmed by a detailed neurootological examination including otoscopy, stapedius reflexes, middle ear pressure measurements, pure tone audiometry, tinnitus pitch and loudness matches. For the present study, those patients with a history of vestibular schwannoma, Menière's disease, or pathological middle ear conditions were excluded. The remaining subjects suffered from primary tinnitus and family histories were available in 139 subjects. Of these, 38% reported at least one first-degree relative affected by tinnitus. Tinnitus severity was assessed by the Tinnitus Questionnnaire (TQ) [21] in 283 patients (98.3%). All participating subjects were Caucasians and most originated from the Upper Palatinate region of Bavaria. Nine external control populations, matched for Caucasian background, served to test for association of *KCNE3 *variants with tinnitus susceptibility ([22-25], PharmGKB, dbSNP, HapMap-CEU and HapMap-TSI populations). The level of statistical significance was set at *p *< 0.05.

Genomic DNA was extracted from lymphocytes using standard pocedures prior to amplification of the *KCNE3 *coding region by PCR. Briefly, a 748 bp amplicon was generated using the following oligomers: 5'-CCA TCC CCT CTC TCT TTT CT-3' (forward) and 5'-CCA GAG CAT CTT CCT GTC TC-3' (reverse). PCR products were purified for Sanger sequencing and for the identification of variants against the human genome reference (Genome Reference Consortium Build 37, February 2009 release). Multiple sequence alignments were conducted with DNA Dynamo 1.0 (Blue Tractor Software, UK). STATA 8.0 (Stata Corporation, College Station, TX, USA) was used for statistical analyses. *KCNE3 *allele frequencies from reference populations were compared to the present data using Fisher's exact test. All *p *values are uncorrected for multiple testing.

For assessing the functionality of coding variants observed in our sample, evolutionary conservation was assessed with a phylogenetic hidden Markov model-based method, PhastCons, that describes the process of DNA substitution at each site in a genome and the way this process changes from one site to the next [26]. Genomic sequences from 46 placental mammals were aligned to the human reference delimited by forward and reverse primers using a Threaded Blockset Aligner [27] as implemented in the conservation track of the UCSC Genome Browser [28]. Linkage disequilibrium and conformity with Hardy-Weinberg equilibrium was measured with HaploView 4.2. [29] and PS V2.1.15 [30] was used for power simulations.

We confirmed two known coding variants with observed heterozygosities of 0.215 (F66F) and 0.01 (R83H), and featuring genotype distributions that conformed to the Hardy-Weinberg equilibrium (p > 0.79, Table 1). Five additional *KCNE3 *variants listed in dbSNP were absent from our sample (rs34604640, rs17215444, rs17221826, rs17221833, rs11822977). Four previously reported *KCNE3 *mutations were also absent: T4A [31], V17M [32], R53H [33], and R99H [31,34]. No novel sequence variants were observed. When minor allele frequencies for F66F and R83H were compared to reference frequencies from nine Caucasian control populations, no significant difference was noted (p > 0.22, Table 2). Power simulations, based on the entire sample of patients diagnosed with chronic tinnitus and on published control data, indicated that we should expect a statistical power of >80% to detect a susceptibility factor with an allelic relative risk of >1.56 for the F66F variant, and of >3.27 for the R83H amino acid exchange. The number of tinnitus cases needed to reach this power was estimated at 2,707 and 65,083, respectively.

**Table 1:** Observed allele frequencies for the *KCNE3 *sequence screened in subjects with chronic tinnitus (2N = 576).

SNP (dbSNP ID)	chr11 position	variant amino acid	minor allele frequency in chronic tinnitus	homozygous/heterozygous carriers of the minor allele (p_HWE_)
g.15,002T>C	74,168,599	T4A	0.000	-

g.15,041G>A	74,168,560	V17M	0.000	-

g.15,108C>G (rs34604640)	74,168,493	P39R	0.000	-

g.15,131C>G (rs17215444)	74,168,470	R47G	0.000	-

g.15,150G>A	74,168,451	R53H	0.000	-

**g.15,190T>C (rs2270676)**	**74,168,411**	**F66F**	**0.125**	**5/62 (0.788)**

g.15,220C>T (rs17221826)	74,168,381	I76I	0.000	-

**g.15,240G>A (rs17215437)**	**74,168,361**	**R83H**	**0.005**	**0/3 (0.930)**

g.15,255G>A (rs17221833)	74,168,346	R88H	0.000	-

g.15,288G>A	74,168,313	R99H	0.000	-

g.15,321C>T (rs11822977)	74,168,280	-	0.000	-

Numbering of SNPs refers to RefSeq NG 0118331.1. Polymorphic variants in bold.

**Table 2:** Reference allele frequencies and measures of association for g.15,190T>C (rs2270676 encoding  F66F) and g.15,240G>A (rs17215437 encoding R83H), based on data from 474 and 1,140  Caucasian controls, respectively.

Healthy controls (N_unrelated_)	study	g.15,190T>C (F66F) frequency in controls	g.15,190T>C (F66F) controls vs. tinnitus patients	g.15,240G>A (R83H) frequency in controls	g.15,240G>A (R83H) controls **vs**. tinnitus patients
"White" Brazilian (40)	[23]	-	-	0.000	n.s.

French (506)	[24]	-	-	0.008	n.s.

German (321)	[25]	-	-	0.005	n.s.

U.S., European descent (180)	[22]	0.117	n.s.*	0.003	n.s.

Caucasian (48)	PharmGKB PS203664	0.083	n.s.	0.010	n.s.

Caucasian (45)	dbSNP ss65626119	0.089	n.s.	-	-

Caucasian (45)	dbSNP ss65626296	-	-	0.011	n.s.

Utah residents with Northern and Western European ancestry from the CEPH collection (113)	HapMap-CEU ss38798969	0.093	n.s.	-	-

Tuscans in Italy (88)	HapMap-TSI ss38798969	0.108	n.s.	-	-

dbSNP data refer to NCBI build 132, HapMap data refer to two  out of the 11 populations in phase 3.

*n.s. = not significant

**Figure 1:**
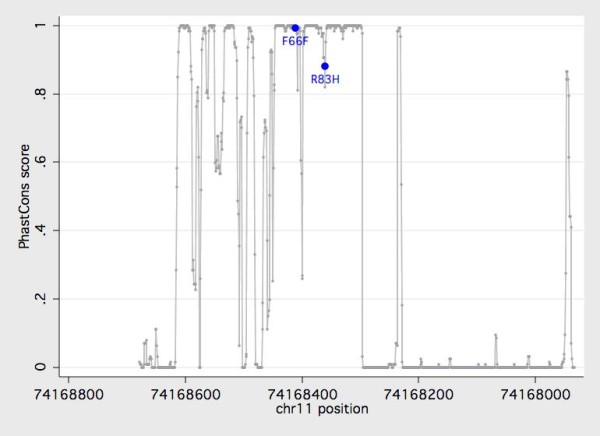
**Evolutionary conservation of the KCNE3 amplicon under study**. The degree of conservation (PhastCons score) is plotted against the physical position based on genomic sequence information from 46 placental mammals. Both g.15,190T>C (rs2270676 encoding F66F) and g.15,240G>A (rs17215437 encoding R83H) map to a highly conserved part of the open reading frame. The ORF is delimited by positions 74,168,608 and 74,168,296 on the February 2009 Homo sapiens high coverage assembly (Hg19) from the Genome Reference Consortium (GRCh37).

We next examined whether *KCNE3 *variants could serve as predictors of tinnitus severity in the population under study. Overall, TQ scores averaged 40.0 ± 18.4 (mean ± SD) out of 84 points (N = 283). By this measure, tinnitus was rated mild (0 to 30 points) in 94 subjects (33.2%), moderate (31 to 46 points) in 84 subjects (29.7%), severe (47 to 59 points) in 55 subjects (19.4%), and extreme (60 to 84 points) in 50 subjects (17.7%). There was no significant difference in mean TQ scores between carriers and non-carriers of the minor alleles at either of the two confirmed polymorphic *KCNE3 *nucleotides (p > 0.22, data not shown). As we encountered only three carriers of the rare missense variant R83H (f_HIS83 _= 0.005), the impact of this substitution on tinnitus severity could not be fully judged. However, neither of the two variants is expected to be inert by comparative genomic analysis (Figure 1). More detailed examinations, e.g. by heterologous expression, are required to understand the effects of F66F and R83H on potassium signaling.

The above evidence illustrates that the *KCNE3 *coding region is remarkably well conserved in a moderately sized population with chronic tinnitus, evoking similar findings in other pathologies [35,36]. The power of the present study is, however, inadequate to rule out an association with tinnitus. Considering the phenotypic overlap of tinnitus and Menière's disease, the present negative findings diverge from the significant association of *KCNE3 *with Menière's disease claimed by Doi et al. [9], but this may be due to methodological issues [22]. The possibility remains that among our external control subjects classified as «healthy», some may have experienced mild forms of tinnitus. Future studies employing a matched set of cases and controls should put this into perspective. Moreover, we gave priority to the gene's coding region and did not adress regulatory variation, or variation in the remaining noncoding regions. Results of the present screening are therefore preliminary with regard to a proposed functionality of *KCNE3 *in tinnitus.

A complex interplay of multimeric potassium channel-forming proteins in auditory perception calls for follow-up examinations of interacting molecules that control the excitability of sensory neurons, including structures that are targeted by anti-tinnitus drugs. For lidocaine, these candidates comprise KCNA1 and KCNC1, plus KCNH2 [37,38], which has also been implicated in phenytoin effects [39].

In view of the limited power of our pilot study and the need to assesss promotor variation, more research is invited to address *KCNE3 *impact on the perception of phantom auditory sensations. Finally, variation relating to channel structures that interact with *KCNE3 *may also help in predicting the response to membrane-stabilizing drugs. *KCNE3 *encodes a beta transmembrane subunit that assembles with several alpha subunits to modify gating and pharmacological sensitivity. It is highly likely that a mutation in *KCNE3 *alone may not be indicative of tinnitus, but when mutations are present in both *KCNE3 *and the channel with which it is interacting (e.g. KCNQ1) the biophysical properties of the channel complex are significantly altered.

## Competing interests

The authors declare that they have no competing interests.

## Authors' contributions

Authors PGS, BL and TK acquired, analysed and interpreted the data. PGS designed the study and drafted the original manuscript. All authors revised the manuscript for important intellectual content and provided final approval of the version to be published.
